# QSAR Studies on Andrographolide Derivatives as α-Glucosidase Inhibitors

**DOI:** 10.3390/ijms11030880

**Published:** 2010-03-02

**Authors:** Jun Xu, Sichao Huang, Haibin Luo, Guoji Li, Jiaolin Bao, Shaohui Cai, Yuqiang Wang

**Affiliations:** 1 Pharmacy College, Jinan University, Guangzhou, 510632, China; E-Mails: goldstar_8209@163.com (J.X.); sichaohuang.cn@gmail.com (S.H.); 1027559485@qq.com (G.L.); 23854695@qq.com (J.B.); 2 School of Pharmaceutical Sciences, Sun Yat-sen University, Guangzhou, 510275, China; E-Mail: luohb77@mail.sysu.edu.cn (H.L.)

**Keywords:** andrographolide, QSAR, α-glucosidase, HQSAR

## Abstract

Andrographolide derivatives were shown to inhibit α-glucosidase. To investigate the relationship between activities and structures of andrographolide derivatives, a training set was chosen from 25 andrographolide derivatives by the principal component analysis (PCA) method, and a quantitative structure-activity relationship (QSAR) was established by 2D and 3D QSAR methods. The cross-validation *r*^2^ (0.731) and standard error (0.225) illustrated that the 2D-QSAR model was able to identify the important molecular fragments and the cross-validation *r*^2^ (0.794) and standard error (0.127) demonstrated that the 3D-QSAR model was capable of exploring the spatial distribution of important fragments. The obtained results suggested that proposed combination of 2D and 3D QSAR models could be useful in predicting the α-glucosidase inhibiting activity of andrographolide derivatives.

## 1. Introduction

*Andrographis paniculate* is a plant widely used as a traditional Chinese medicine in China, India, and other Asian countries [1,2]. Extracts and constituents of *Andrographis paniculate* exhibit broad pharmacological activities, such as anti-bacterial, ant-malarial, anti-inflammatory, anti-tumor, immunological regulation, and hepatoprotective effects [3–12]. Lately, some andrographolide derivatives were reported to decrease blood glucose level by inhibiting α-glucosidase [13,14]. It has been well known that α-glucosidase is a key enzyme in the absorption of sugar in the small intestine mucous membrane, and its activity is closely related to blood glucose levels. Studies also indicated that α-glucosidase might be involved in diabetes [15–20]. Accordingly, α-glucosidase is considered an important target for the design of antidiabetic drugs. Recently, efforts had been made in modification and synthesis of novel andrographolide derivatives to find more potent and safer α-glucosidase inhibitors. Knowledge about the relationships between structures of andrographolide derivatives and their inhibitory activities on α-glucosidase could greatly facilitate the drug discovery process.

QSAR [21] has been widely used for years to provide quantitative analysis of structure and activity relationships of compounds. Statistical methods are applied in QSAR modeling to establish correlations between chemical structures and their biological activities. Once validated, the findings can be used to predict activities of untested compounds. Recently, computer-assisted drug design based on QSAR has been successfully employed to develop new drugs for the treatment of cancer, AIDS, SARS, and other diseases [22–29]. With the availability of large commercial databases and highly efficient programs including Sybyl, Discovery studio, MOE and so on, it is estimated that QSAR modeling as a tool could remarkably reduces the cost of drug discovery [30].

In this study, 2D QSAR models were constructed to describe the important fragments in andrographolide derivatives and 3D QSAR models were established to explore the spatial distribution of important groups. The combination of 2D and 3D QSAR models could better summarize the QSAR of andrographolide derivatives in inhibiting α-glucosidase.

## 2. Computational Methods

### 2.1. Database and Software

**Figure 1.**
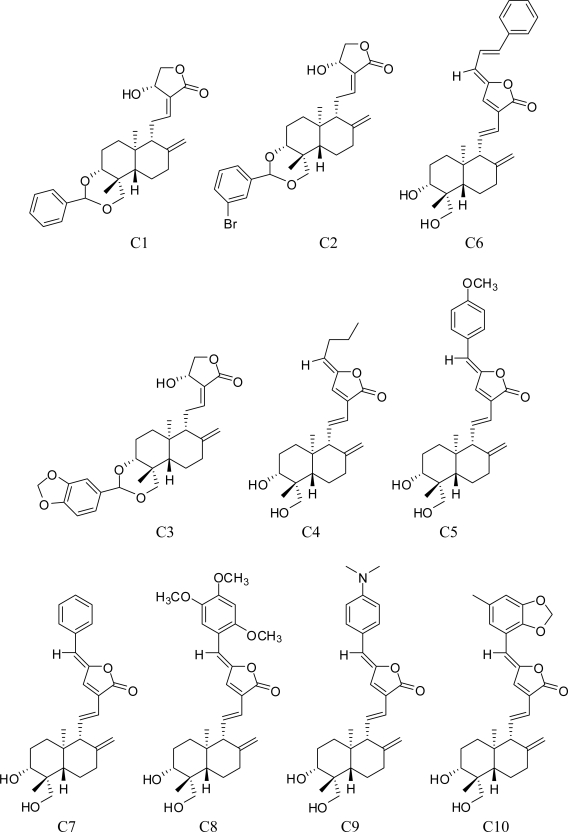

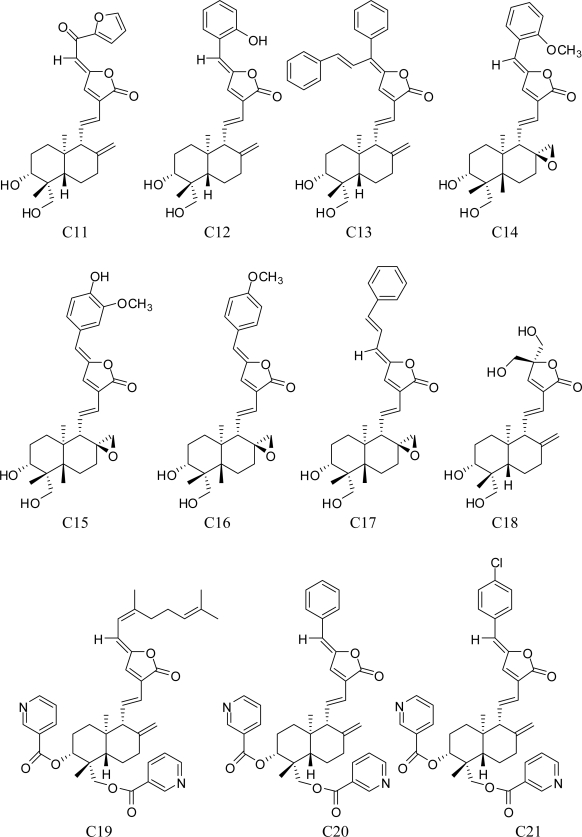

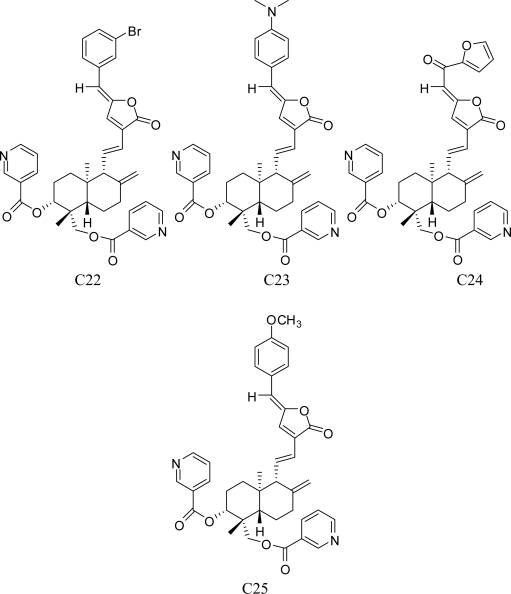
Formulae of the studied andrographolide derivatives.

The structures and inhibitory activities (IC50) of 25 andrographolide derivatives (Figure 1) were collected from the literature, and served as the database to build QSAR models [13,14,31]. PLogIC50 was used as the dependent variable of QSAR model. PCA, HQSAR, CoMFA, CoMSIA were performed by Sybyl7.03 (Tripos Co., LTD) program.

### 2.2. Training Set Selection

Principle Component Analysis (PCA), employed to select the training set, could be applied to explain the differences among the 25 andrographolide derivatives through diversities of the structures’ parameters and to exhibit their distribution on a 2D plot [32]. Furthermore, the most descriptive compounds (MDC) or the largest minimum distance (LMD) methods were applied to select the training set according to the distribution of these compounds.

### 2.3. Generation and Validation of the 2D QSAR Model

Hologram QSAR (HQSAR) offers the ability to rapidly generate QSAR models of high statistical quality and predicted value by SYBYL line notation (SLN), cyclic redundancy check (CRC) and partial least squares (PLS) [33–35]. The premise of HQSAR is that since the structure of a molecule is encoded within its 2D fingerprint and that structure is the key determinant of all molecular properties (including biological activity), it should be possible to predict the activity of a molecule from its fingerprint.

The training set was used to establish 2D-QSAR model by HQSAR, and the best 2D-QSAR model was applied by the criterion of cross-validation R^2^. The test set’s biological activity was predicted by the best 2D-QSAR model, whose predictability was validated by correlation coefficient between the predicted and experimental values. The most common structure (MCS) could be calculated by HQSAR. Based on the MCS of andrographolide derivatives, the contributions of molecules’ fragments to biological activity should be analyzed for describing the QSAR of andrographolide derivatives as α-glucosidase inhibitors.

### 2.4. Generation and Validation of the 3D QSAR Model

The three-D QSAR model applies PLS to explore the relationships between the physicochemical variables and biological activity. Cross-validation is used to estimate the QSAR model’s predictability. In general, a LOO cross-validated coefficient Q^2^ (higher than 0.5) can be considered as statistically high predictive ability [36]. CoMFA, which is widely utilized in 3D-QSAR research, claims that if a group of similar compounds are ligands of the same receptor, their bioactivities depend on the differences of the molecules’ fields surrounding them [37]. CoMFA can exhibit a contour map in a 3D graph, which makes it easier to distinguish differences between compounds with strong and weak activities. CoMSIA is another 3D-QSAR method that adopts a Gaussian function instead of traditional Coulomb and Lennard-Jones’ function used in CoMFA [38]. Therefore, CoMSIA efficiently avoids the shortcomings of CoMFA in which only the steric and electrostatic fields are used. The leave-one-out (LOO) method is employed to validate the predictability of the models and Y-Randomization test is used to validate the robustness of the models [39].

In this study, CoMFA and CoMSIA were both utilized to generate 3D-QSAR models, and then the relative higher predictive 3D-QSAR models were selected by comparison. Subsequently, the selected models were further optimized by the Focusing method [40]. This method describes the different contributions of different grids in CoMFA and CoMSIA to the bioactivities of the compounds by weighting, which was expected to selectively enhance or impair the contributions of different grids and improve the resolution. Moreover, the biological activities of test set were predicted by the optimized QSAR model. The best QSAR model was determined by comparing the parameters of the model and correlation between the predicted and experimental values of the test sets.

## 3. Result and Discussion

### 3.1. Training Set Selection

The selection of the training set is one of the most important steps in QSAR modeling, since the establishment and optimization of a QSAR model are based on this training set. Predictability and applicability of a QSAR model also depend on the training set selection [41,42]. Usually, the compounds serving as the training set should have three characteristics: (1) maximum structural diversity; (2) maximum activity diversity; (3) similarity of interactions [43]. Besides, both molecular structures and biological activities of the test set should be covered by the ranges of the training set. In this research, PCA was applied to select a training set from among 25 andrographolide derivatives. PCA is a statistical technique useful for summarizing all the information encoded in the structures of compounds. It is also very helpful for understanding the distribution of the compounds.

**Figure 2.**
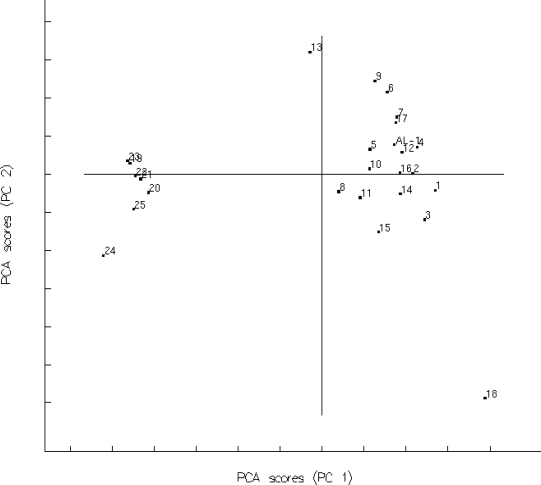
PCA plot for studied compounds **1**–**25**.

The distribution pattern of the 25 andrographolide derivatives is shown in Figure 2. There were different population densities in the Figure. Eighteen compounds (**1**, **3**–**8**, **11**, **13**, **16**–**21** and **23**–**25**) were selected as the raining set by the MDC method. The rest of them (compounds **2**, **9**, **10**, **14**, **15** and **22**) were used as the test set whose biological activities were covered by the training set.

### 3.2. Establishment and Validation of 2D-QSAR Model

The best cross-validation r^2^ (0.731) and standard error (0.225) illustrated that the 2D-QSAR model could be applied to predict the biological activity of andrographolide derivatives as α-glucosidase inhibitors. The predicted and experimental biological activities of andrographolide derivatives are shown in Table 1. The results of the correlation coefficient R^2^, standard error of the training set (0.840, 0.174) and test set (0.949, 0.104) suggested that the 2D-QSAR model could be used to explain the QSAR of andrographolide derivatives as α-glucosidase inhibitors.

**Figure 3.**
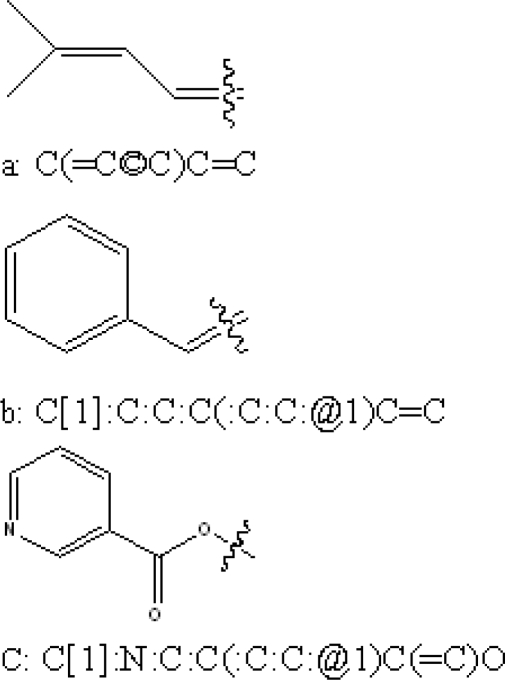
Key fragments of 2D-QSAR Model.

Furthermore, three key fragments (Figure 3) were selected according to PLS coefficient. The predicted activity = $C_{0}+\sum_{i}\left(C_{i}\times b_{i}\right)$ where *C_0_* = the offset, *C_i_* = the PLS coefficient associated with bin I in the hologram, *b_i_*= the number of fragments hashed into bin *i*.

**Figure 4.**
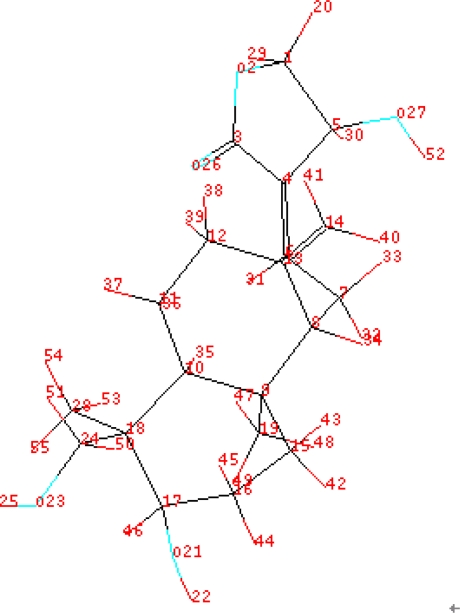
Structure of andrographolide.

The PLS coefficient was the standardization for judging which fragment was the key fragment. The larger the PLS coefficient, the more important the fragment was for andrographolide derivatives’ biological activity. According to the criterion, C (=C©C)C=C or C[1]:C:C:C(:C:C:@1)C=C attached to C_3_ of andrographolide (Figure 4) and C[1]:N:C:C(:C:C:@1)C(=C)O attached to C_17_ of andrographolide were suggested as the key fragments.

### 3.3. Establishment and Validation of the 3D-QSAR Model

**Figure 5.**
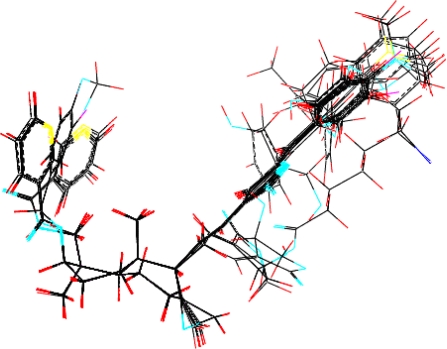
Alignment of the database.

The 18 compounds were energy minimized, added charges and aligned (Figure 5). CoMFA and CoMSIA were used to develop a number of QSAR models based on the properties of compounds belonging to different fields (steric, electrostatic, hydrophobic, H-donor and acceptor, Table 2). Since the QSAR model was employed to predict unknown compounds’ activity, the model’s predictability was the criterion to judge which QSAR model was the best. Predictability of a QSAR model was not only expressed by cross-validation (q^2^) but also by validation of the test set. The results illustrated that four models (**4**, **8**, **10** and **11**) had the top four predictabilities, so the Focus method was then applied to optimize these models, and further improved predictability for model **4**, **10** and **11**, but not for model 8. Among these models (model **8**, **13**, **15** and **16**), model **16** exhibited the best predictability as indicated by the highest Q2 value. Predictability of these models (**8**, **13**, **15** and **16**) was further evaluated using a test set. Model **16** also provided the best prediction with a correlation coefficient R^2^ (0.941) (Table 3). Overall, this model represented the best QSAR model (q^2^ = 0.794, R^2^_cv_ = 0.915, SE_cv_ = 0.127, R^2^_test set_ = 0.941, SE_test set_ = 0.104). Y-Randomization test (q^2^ = 0.199) suggested that the model also had a good robustness. Table 4 showed Comparison between predicted PLogIC50 of database and experimental values by using Model **16**.

**Figure 6.**
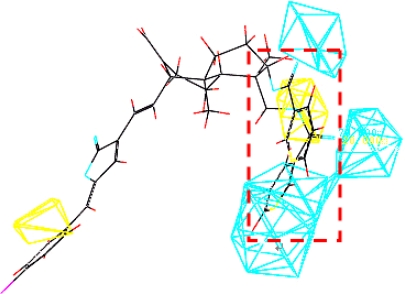
Compound **21** placed in the H-accept contour map.

**Figure 7.**
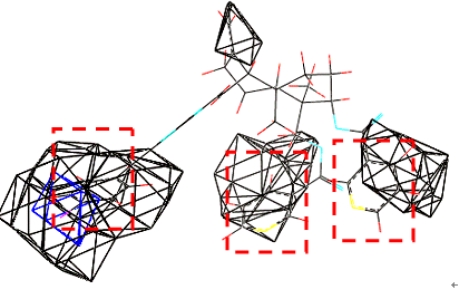
Compound **21** placed in the hydrophobic contour map.

**Figure 8.**
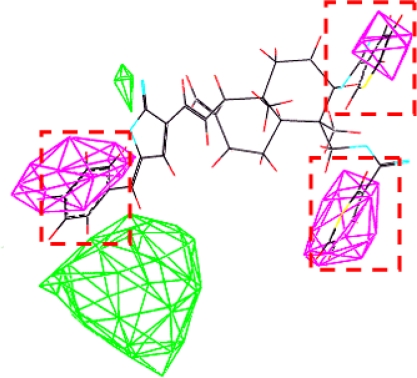
Compound **21** was placed in the steric contour map.

Model **16** used steric field, hydrophobic field and H-acceptor field together to describe the relationship between activities and structures of andrographolide derivatives. H-bond receptive atoms and groups in the region marked by blue lines (Figure 6) were favorable for the activities of the compounds, while the atoms and groups in the region marked by yellow lines impaired the activities. Hydrophobic groups were desirable in the region marked with blue lines but not the region marked by dark lines (Figure 7). In addition, the activities of the andrographolide derivatives were enhanced by the presence of steric groups in the region marked by purple lines instead of the region marked by green lines (Figure 8). The compounds with structures fitting well into the 3D contour maps derived from the model **16** usually exhibited potent inhibitory activity (e.g., compounds **20**, **21**, **22** and **23**). In contrast, weak inhibitors such as compounds **3**, **4**, **13** and **16** did not have a good fit to the 3D contour maps.

Compound **21** (potent α-glucosidase inhibitor PLogIC_50_ = 5.222) was layed in the 3D contour maps of model **16** to illustrate the key groups (marked by red dashed lines in Figures 5, 6, and 7) correlating with biological activity. C[1]:N:C:C(:C:C:@1)C(=C)O was a key group in all the 3D contour maps (steric, H-accept, hydrophobic) and C[1]:C:C:C(:C:C:@1)C=C was a key group in both steric and hydrophobic 3D contour maps. Both the groups were also calculated as key groups in HQSAR. Combining the results of HQSAR and CoMSIA, the two groups were considered as the key groups associated with biological activity and the result can also be used to screen potent α-glucosidase inhibitors from various databases by virtual screening.

## 4. Conclusions

In our research, 2D QSAR and 3D QSAR models have been successfully established to quantitatively describe the relationship between structures and activities of andrographolide derivatives as α-glucosidase inhibitors. The 2D QSAR model was based on the atomic connection of molecules and suggested that there might be three key groups associated with biological activity. Furthermore, the 3D QSAR model was based on molecular properties belonging to steric, hydrophobic and H-acceptor fields and indicated that compounds with structures fitting better into the 3D contour maps of model 16 had more potent activities. Combining 2D and 3D QSAR models, the key fragments and their spatial distribution could be efficiently identified. The convinced predictability of the model was demonstrated not only by internal validation but also by external validation using a test set. Overall, these results suggested that the developed QSAR model could be used to predict the inhibitory activities of unknown andrographolide derivatives on α-glucosidase. Application of this model would greatly facilitate the discovery of better α-glucosidase inhibitors.

## Figures and Tables

**Table 1.** Comparison of the predicted PLogIC50 of database with the experimental values by using 2D-QSAR Model.

**Compound**	**ACT^a^**	**PRE^b^**	**|Δ|^c^**	**Compound**	**ACT**	**PRE**	**|Δ|**
1	4.000	3.933	0.067	2	4.000	3.995	0.05
3	3.959	3.876	0.109	4	3.959	4.054	0.095
5	-	-	-d	6	4.237	4.139	0.098
7	4.237	4.159	0.078	8	4.076	4.087	0.011
9	4.155	4.061	0.094	10	4.000	4.099	0.099
11	4.000	4.089	0.089	12	-	-	-d
13	3.959	4.176	0.217	14	4.000	3.946	0.054
15	3.983	3.924	0.059	16	3.921	3.961	0.040
17	3.996	3.954	0.042	18	3.971	3.902	0.069
19	4.553	4.686	0.133	20	4.796	4.813	0.017
21	5.222	4.806	0.416	22	4.854	4.798	0.056
23	4.602	4.715	0.113	24	4.444	4.745	0.301
25	4.959	4.698	0.261				

a: Experimental data (PLogIC_50_)

b: Predicted data (PLogIC_50_)

c: |a–b|

d: Outline compounds.

**Table 2.** Comparison of different 3D-QSAR models.

**No.**	**Method**	**Field^a^**	**OC^b^**	**(q^2^)^c^**	**SE^d^**	**(R^2^)^e^**	**F**
1	CoMFA	S+E	1	0.741	0.178	0.819	67.905

2		S	2	0.748	0.159	0.866	45.280
3		E	1	0.710	0.187	0.802	60.592
4		H	2	0.771	0.132	0.907	68.505
5		D	1	0.313	0.297	0.498	14.876
6		A	1	0.724	0.184	0.807	62.902
7		S+E	1	0.732	0.182	0.812	64.778
8	CoMSIA	S+H	1	0.774	0.148	0.875	105.050
9		S+A	2	0.738	0.159	0.866	45.251
10		S+E+H	1	0.755	0.169	0.838	77.788
11		S+H+A	2	0.759	0.130	0.910	70.509
12		S+E+H+A	1	0.747	0.174	0.829	72.588
13^f^		H(Focus)	1	0.776	0.144	0.882	112.028
14^f^		S+H(Focus)	2	0.772	0.1.43	0.891	57.188
15^f^		S+E+H(Focus)	2	0.763	0.148	0.884	53.422
16^f^		S+H+A(Focus)	2	0.794	0.127	0.915	75.093
	Y-Random	S+H+A(Focus)	1	0.199	-	-	-

a: S: Steric field, E: Electrostatic field, H: Hydrophobic field.

D: H-donor field, A: H-acceptor field.

b: Optimum of component.

c: The models’ cross-validation r^2^.

d: Standard Error.

e: Correlation coefficient between predicted and experimental PLogIC50 of 18 compounds.

f: The model was optimized by Focus Method.

**Table 3.** Correlation coefficient between predicted and experimental PLogIC50 of the test set by model **13**, **8**, **15**, and **16.**

**No.**	**Models**	**R^2^**	**Slope**	**SE**
13	H(Focus)	0.906	1.007	0.143
8	S+H	0.927	0.974	0.121
15	S+E+H(Focus)	0.895	0.937	0.142
16	S+H+A(Focus)	0.941	0.933	0.104

**Table 4.** Comparison between predicted PLogIC50 of database and experimental values by using Model **16**.

**Compound**	**ACT^a^**	**PRE^b^**	**|Δ|^c^**	**Compound**	**ACT**	**PRE**	**|Δ|**
1	3.996	3.960	0.04	2	4.000	3.960	0.04
3	3.959	3.970	0.011	4	3.959	3.999	0.04
5	-	-	-d	6	4.237	4.238	0.001
7	4.237	4.204	0.033	8	4.076	4.016	0.06
9	4.155	4.179	0.029	10	4.000	4.119	0.119
11	4.000	3.935	0.065	12	-	-	-
13	3.959	4.111	0.152	14	4.000	4.150	0.150
15	3.983	4.112	0.129	16	3.921	4.075	0.154
17	3.996	3.916	0.08	18	3.971	3.903	0.068
19	4.553	4.621	0.068	20	4.796	4.863	0.068
21	5.222	5.067	0.155	22	4.854	4.886	0.032
23	4.602	4.831	0.229	24	4.444	4.481	0.037
25	4.959	4.698	0.261				

a: Experimental data (PLogIC_50_)

b: Predicted data (PLogIC_50_)

c: |a–b|

d: Outline compounds
